# Signal regulatory protein beta 2 is a novel positive regulator of innate anticancer immunity

**DOI:** 10.3389/fimmu.2023.1287256

**Published:** 2023-12-05

**Authors:** Nienke Visser, Levi Collin Nelemans, Yuan He, Harm Jan Lourens, Macarena González Corrales, Gerwin Huls, Valerie R. Wiersma, Jan Jacob Schuringa, Edwin Bremer

**Affiliations:** Department of Hematology, University of Groningen, University Medical Center Groningen (UMCG), Groningen, Netherlands

**Keywords:** SIRP-ß2, cd47, phagocytosis, macrophage, antigen presentation

## Abstract

In recent years, the therapeutic (re)activation of innate anticancer immunity has gained prominence, with therapeutic blocking of the interaction of Signal Regulatory Protein (SIRP)-α with its ligand CD47 yielding complete responses in refractory and relapsed B cell lymphoma patients. SIRP-α has as crucial inhibitory role on phagocytes, with e.g., its aberrant activation enabling the escape of cancer cells from immune surveillance. SIRP-α belongs to a family of paired receptors comprised of not only immune-inhibitory, but also putative immune-stimulatory receptors. Here, we report that an as yet uninvestigated SIRP family member, SIRP-beta 2 (SIRP-ß2), is strongly expressed under normal physiological conditions in macrophages and granulocytes at protein level. Endogenous expression of SIRP-ß2 on granulocytes correlated with trogocytosis of cancer cells. Further, ectopic expression of SIRP-ß2 stimulated macrophage adhesion, differentiation and cancer cell phagocytosis as well as potentiated macrophage-mediated activation of T cell Receptor-specific T cell activation. SIRP-ß2 recruited the immune activating adaptor protein DAP12 to positively regulate innate immunity, with the charged lysine 202 of SIRP-ß2 being responsible for interaction with DAP12. Mutation of lysine 202 to leucine lead to a complete loss of the increased adhesion and phagocytosis. In conclusion, SIRP-ß2 is a novel positive regulator of innate anticancer immunity and a potential costimulatory target for innate immunotherapy.

## 1 Introduction

Development of anticancer immunity critically depends on the interplay between the innate and adaptive immune system. One of the most important mechanisms of the innate immune system to facilitate cancer immunity is phagocytic uptake of target cells by phagocytes, such as macrophages and dendritic cells, and the subsequent presentation of antigens to the adaptive immune system (1). Phagocytosis is, among others, regulated by the signal regulatory protein (SIRP)-family.

The SIRP-family is a paired receptor family with an important immunoregulatory function in innate immunity that comprises of inhibitory, stimulatory and non-signaling receptors (2). The most prominent SIRP family member is the inhibitory protein SIRP-alpha (SIRP-α), which binds to its ligand CD47. The CD47/SIRP-α axis is a well-established innate immune checkpoint, in which overexpression of CD47 on cancer cells triggers SIRP-α-mediated inhibition of cancer cell phagocytosis (3). Herewith, removal and immunogenic processing of cancer cells by macrophages and dendritic cells is limited. In line with this, high CD47 expression is associated with poor clinical prognosis in various malignancies (4–7).

Based on these features, the CD47/SIRP-α axis has become a prominent therapeutic target in cancer. Indeed, combination therapy of a CD47 antagonist with positive phagocytic stimulators such as the monoclonal antibody Rituximab (RTX) yielded complete responses in lymphoma patients refractory to RTX (8). Further, combination with the epigenetic drug azacytidine has yielded prominent clinical activity in Myelodysplastic Syndrome (9). In contrast, monotherapy with antagonistic antibodies has yielded few clinical responses (10), suggesting the balance between anti-phagocytic and pro-phagocytic signals is not sufficiently disturbed. These findings clearly highlight the therapeutic relevance of innate immunoregulatory processes for activating functional anti-cancer immunity.

In recent years, additional negative as well as positive regulators on innate anti-cancer immunity have been identified. For example, members of the leukocyte immunoglobulin-like receptor B (LILRB) family are known to inhibit signals in macrophages (11). Expression of LILRB1 on macrophages inhibits phagocytosis of cancer cells upon CD47 antibody treatment, an effect reversed by inhibition of LILRB1 (12). In addition, expression of LILRB2 on macrophages also inhibits phagocytosis of cancer cells. JTX-8064, a highly specific and potent blocker of this LILRB member, reprograms macrophages from an immunosuppressive to an immunostimulatory phenotype and thereby stimulates and activates T cells (11). Reversely, expression of the pro-phagocytic receptor SLAMF7 was reported to be crucial for execution of macrophage-mediated phagocytosis of cancer cells (13), although cancer cell-expression of SLAMF7 is not required for CD47 antibody treatment (13). Another example of a pro-phagocytic receptor is the G protein-coupled receptor (GPCR), specifically GPR84. GPR84 is found to be upregulated in AML leukemic stem cells and expression of this GPR member is correlated with significantly overall survival of patients diagnosed with acute myeloid leukemia (AML) (14, 15). In this context it is worthwhile to note that several of the other much less researched SIRP family members may also be of therapeutic interest. For instance, SIRP-γ is an immunostimulatory receptor expressed on T cells and its inhibition using an anti-SIRP-γ antibody downregulated IFN-gamma secretion and ameliorated Graft versus Host Disease (16). Further, although the exact role of SIRP-beta 1 (SIRP-ß1) is as yet unknown, this family member is expressed on myeloid cells and can trigger costimulatory signaling through the immunoreceptor tyrosine-based activation motif (ITAM)-containing adaptor protein DAP12. Specifically, SIRP-ß1 has a charged lysine residue in the transmembrane domain responsible for recruiting DAP12 (2). DAP12 is involved in immunostimulatory signaling and plays a central role in an extended array of receptors in NK cells, granulocytes, monocytes/macrophages and DCs (17). Finally, the family member SIRP-beta 2 (SIRP-ß2) has so far not been investigated, with only the full-length cDNA encoding SIRP-ß2 being reported to date (18).

Here, we investigated SIRP-ß2 and delineated its expression patterns, its immunostimulatory signaling pathways and its biological role in innate immune responses against cancer *in vitro*. Our results position SIRP-ß2 as a novel positive regulator of innate anticancer immunity and as a possible new clinically relevant member of the SIRP-family.

## 2 Results

### 2.1 SIRP-ß2 is expressed in myeloid effector cells

**Figure 1:**
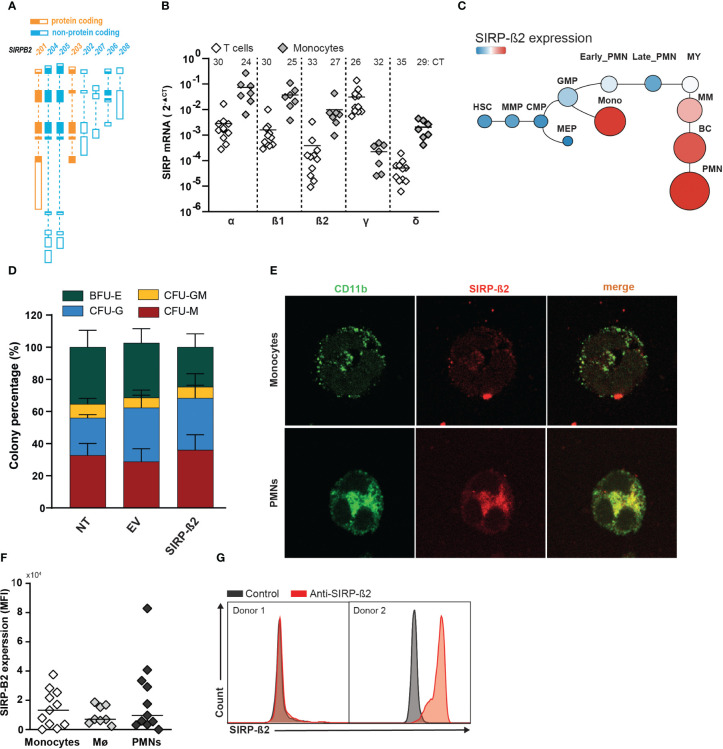
Expression pattern, genomic arrangement and transcript variants of the SIRP family. **(A)** Schematic representation of the protein coding and non-coding transcript variants of the SIRP-ß2 locus. **(B)** Quantitative RT-qPCR analysis of mRNA expression in T cells and monocytes of SIRP family transcripts. **(C)** mRNA SIRP-ß2 expression in hierarchical differentiation tree. **(D)** Colony percentage of BFU-E (Burst Forming Unit E), CFU-GM (Colony-Forming Unit for granulocytes and macrophages) after 14days. 1x10^3^ CD34+ cells derived from cord blood (CB) plated, transduced with NT, EV and SIRP-ß2. **(E)** Confocal images of the surface expression of CD11b and SIRP-ß2 on monocytes. **(F)** Flow cytometry of SIRP-ß2 levels on monocytes (n=11), monocyte-derived macrophages (n=8) and polymorphonuclear neutrophil (PMN) (n=11), including different donors. **(G)** SIRP-ß2 expression pattern on flow cytometry from 2 different donors.

The SIRP family is located on chromosome p13 and comprises of the members SIRP-α, SIRP-ß1, SIRP-γ, the soluble member SIRP-δ, and the as yet uninvestigated member SIRP-ß2. The human SIRP-ß2 locus encodes for 8 different transcript variants of which SIRP-ß2-201 and SIRP-ß2-203 are predicted to be protein coding (**Figure 1A**). The SIRP-ß2-201 variant encodes for a long isoform and SIRP-ß2-203 for a short isoform of SIRP-ß2. Using RT-qPCR, SIRP-ß2 mRNA expression levels in primary T cells and monocytes were investigated along with the other SIRP family members, with SIRP-ß2 being detected in monocytes (**Figure 1B**, 2^-ΔCT^ of 1.0x10^-2^ (range 3.5x10^-2^ – 1x10^-3^). Furthermore, mRNA of SIRP-ß2 as well as SIRP-α and SIRP-ß1 were more highly expressed in monocytes than in T cells of healthy donors (2^-ΔCT^ of 7.0x10^-2^ vs 2.7x10^-3^, 3.5x10^-2^ vs 1.6x10^-3^ vs, and 1.0x10^-2^ vs 3.5x10^-4^ respectively) (**Figure 1B**). On the other hand, SIRP-γ was detected at higher levels in T cells (**Figure 1B**, 2^-ΔCT^ of 3.1x10^-2^ vs 3x10^-4^, respectively), in line with previous reports (16, 19, 20). Expression of SIRP-ß2 mRNA, like SIRP-α and SIRP-β1, was higher in the differentiated myeloid lineage compared to hematopoietic stem cells, particularly in monocytes and polymorphonuclear leukocytes (PMNs), as found on BloodSpot, a database of mRNA expression of hematopoietic cells (**Figure 1C** and **Supplementary Figure 1A**) (21). Furthermore, ectopic expression of SIRP-ß2 in umbilical cord blood (CB) and/or adult CD34+ hematopoietic stem and progenitor (HSPC) cells did not impact colony formation in a CFU assay compared to Empty Vector (EV) and non-transduced (NT) controls (**Figure 1D**; **Supplementary Figure 1B**). Thus, SIRP-ß2 expression was associated with, but did not drive myeloid differentiation.

Expression of SIRP-ß2 at the protein level was confirmed in monocytes, PMNs and monocyte-derived macrophages. SIRP-ß2 co-localized with the plasma membrane protein CD11b for PMNs (R^2 =^ 0.79), macrophage type 0 (R^2 =^ 0.73) and macrophage type 1 (R^2 =^ 0.81) and had a weak co-localization on monocytes (R^2 =^ 0.23) (**Figure 1E**; **Supplementary Figure 1C**). In line with these results, endogenous expression of SIRP-ß2 was detected on a subset of donors in monocytes, monocyte-derived macrophages as well as PMNs, using flow cytometry (**Figures 1F, G**). Taken together, SIRP-ß2 was endogenously expressed in various myeloid effector cell types in a subset of healthy donors on the cell surface.

### 2.2 Ectopic expression of SIRP-ß2 triggered adhesion and facilitated macrophage and granulocyte differentiation

**Figure 2:**
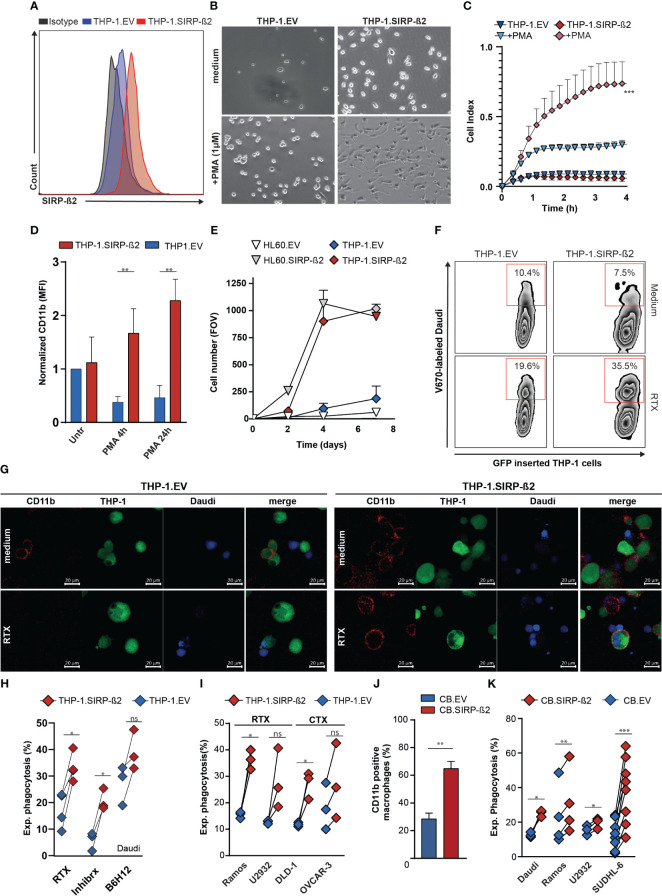
Ectopic expression of SIRP-ß2 in hematopoietic cancer cell lines augments adhesion and their macrophage-mediated phagocytosis. **(A)** Expression of SIRP-ß2 on THP-1.EV and THP-1.SIRP-ß2 cells determined by flow cytometry. **(B)** Representative pictures of adherend THP-1.EV and THP-1.SIRP-ß2, with and without PMA treatment. **(C)**. Quantitative real-time cell adhesion analysis of THP-1.EV and THP-1.SIRP-ß2 upon stimulation with PMA, using Xcelligence. **(D)** CD11b expression of THP-1.EV and THP-1.SIRPß2 after PMA stimulation (n=3). **(E)** Quantitative adherent cells in field of view (FOV) of THP-1.EV/HL60.EV and THP-1.SIRP-ß2/HL60.SIRP-ß2. **(F)** Illustrative flow cytometry data pictures of THP-1 mediated phagocytosis in co-culture with Daudi cells with RTX (1µg/ml), for 3 h **(G)** Representative pictures of THP-1 mediated phagocytosis incubated with Daudi cells for 3 h, treated with RTX (1 µg/ml). **(H)** Quantitative experimental THP-1 mediated phagocytosis of Daudi cells in combination with RTX (1 µg/ml), Inhibrx (1 µg/ml) and B6H12 (1 µg/ml) (n=4). **(I)** Quantitative THP-1 mediated phagocytosis of Ramos and U2932 (+ RTX, 1 µg/ml) and DLD-1 and OVCAR-3 (+ CTX, 1 µg/ml) (n=3). **(J)** CD11b staining on CB derived EV/SIRP-ß2 macrophage (n=3). **(K)** Quantitative CB-derived macrophage phagocytosis of Daudi, Ramos, U2932 and SUDHL-6, incubation 3 h. p values are indicated as: *** p < 0.001, ** p < 0.01, and * p < 0.05. ns (not significant).

To evaluate the functional impact of SIRP-ß2 in myeloid biology, specifically in monocytes and granulocytes, the monocytic cell line THP-1 and granulocytic cell line HL-60 were engineered to overexpress SIRP-ß2-203, as verified using flow cytometry and RT-qPCR (**Figure 2A**; **Supplementary Figure 1D**). Bright field microscopy showed that THP-1.SIRP-ß2-203 (from now on called: THP-1.SIRP-ß2) acquired a more adherent phenotype with or without PMA stimulation, compared to EV transduced (THP-1.EV) cells (**Figure 2B**). Furthermore, THP-1.SIRP-ß2 acquired a more differentiated morphology. This increased adhesion was confirmed using real-time cell analysis (RTCA), yielding rapid and significantly increased adhesion of THP-1.SIRP-ß2 (cell index of 0.6) compared to THP-1.EV (cell index of 0.3) in the first four hours of PMA treatment (**Figure 2C**). In line with this, cell surface expression of CD11b, which is involved in cell adhesion, significantly increased in THP-1.SIRP-ß2 cells stimulated with PMA compared to EV (~4 and ~5 fold increase after four and 24 hours, respectively) (**Figure 2D**). Analogously, ectopic expression of SIRP-ß2 in HL-60 cells increased adhesion of HL-60.SIRP-ß2 compared to HL-60.EV at the same level as THP-1.SIRP-ß2, in combination with PMA (**Figure 2E**). Thus, SIRP-ß2 expression enhanced the differentiation from monocytes towards macrophages.

Ectopic expression of SIRP-ß2 in THP-1 cells did not increase baseline phagocytic activity, with uptake of Daudi cells by differentiated THP-1.EV or THP-1.SIRP-ß2 being comparable (10.4% vs 7.5%, respectively) (**Figure 2F**). However, treatment with the CD20 antibody Rituximab (RTX) did increase phagocytic uptake by 28% for THP-1.SIRP-ß2 and only 9% for THP-1.EV (**Figure 2F**). The occurrence of phagocytosis was visually confirmed with confocal fluorescent microscopy (**Figure 2G**; **Supplementary Figure 1E**). Upon further quantification, THP-1.SIRP-ß2 indeed significantly increased experimental phagocytosis (delta percentage of phagocytosis between treated and medium condition) of Daudi cells upon treatment with RTX, compared to THP-1.EV (**Figure 2H**, RTX). Similarly, experimental phagocytosis of Daudi cells upon treatment with CD47 antibodies Inhibrx or B6H12 was significantly increased in THP-1.SIRP-ß2 compared to THP-1.EV (**Figure 2H**). Furthermore, RTX-treatment potentiated phagocytosis by THP-1.SIRP-ß2 compared to THP-1.EV to a similar degree in lymphoma cell lines Ramos and U2932. Additionally, THP-1.SIRP-ß2 also potentiated phagocytosis of carcinoma cell lines DLD-1 and OVCAR-3 compared to THP-1.EV upon treatment with EGFR-antibody cetuximab (CTX) (**Figure 2I**). Similar results were obtained with CB-derived primary macrophages ectopically expressing SIRP-ß2, with a significant increase in CD11b expression (**Figure 2J**) and a significant increase in phagocytosis of various B cell lymphoma cell lines (p<0.05 for Daudi, U2932; p<0.01 for Ramos; p<0.001 for SUDHL-6) compared to EV-transduced macrophages (**Figure 2K**). No substantial differences were observed between phagocytic uptake of Ramos and SUDHL-6 cells with CB-derived macrophages overexpressing SIRP-ß2.203 (short isoform) or SIRP-ß2.201 (long isoform) and further experiments were continued with the short isoform (**Supplementary Figure 1F**).

In conclusion, SIRP-ß2 promoted cell adhesion and facilitated monocyte and granulocyte adhesion and differentiation of THP-1 and CB-derived macrophages, increased CD11b expression, and increased macrophage-mediated phagocytosis of cancer cells upon antibody-mediated opsonization.

### 2.3 Expression of endogenous SIRP-ß2 on polymorphonuclear leukocytes correlated with enhanced trogocytosis of various tumor cells lines

**Figure 3:**
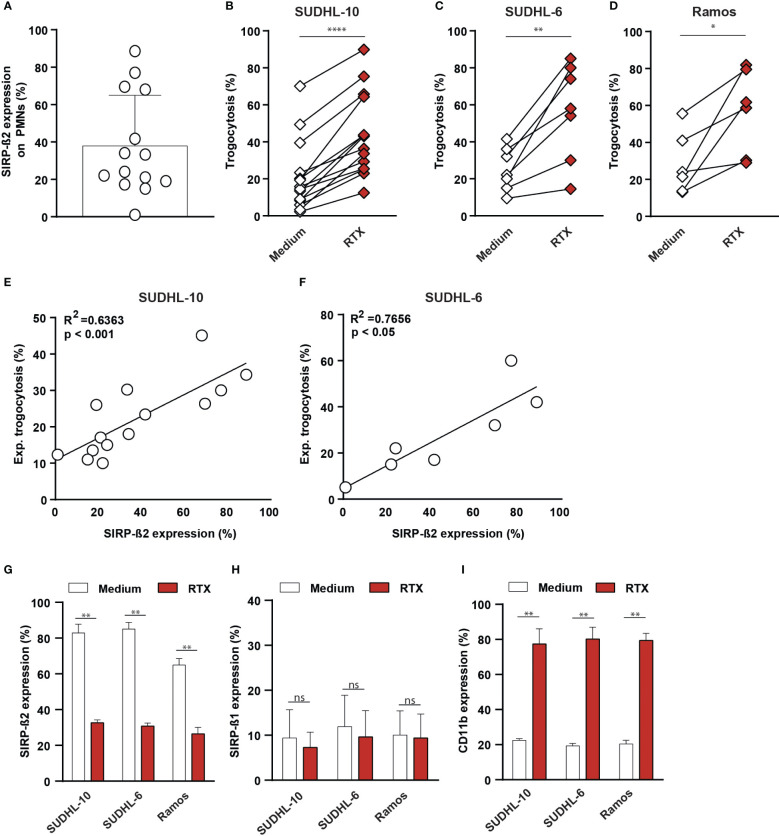
Endogenous SIRP-ß2 on polymorphonuclear leukocytes (PMNs) enhanced trogocytosis of various tumor cells lines which correlates with SIRP-ß2 expression. **(A)** Endogenous SIRP-ß2 expression on PMNs from various individuals (n=14). **(B)** PMN trogocytosis of SUDHL-10 (n=14), **(C)** SUDHL-6 (n=7), **(D)** Ramos (n=5), treated with RTX (1 µg/ml), for 3 h **(E, F)** Correlation of SIRP-ß2 expression (%) and experimental trogocytosis of SUDHL-10 (R^2 =^ 0.6363) and SUDHL-6 (R^2 =^ 0.7656). **(G–I)** Expression of SIRP-ß2, SIRP-ß1 and CD11b measured after 3h trogocytosis of SUDHL-10, SUDHL-6 and Ramos treated with and without RTX (1 µg/ml), by flow cytometry (n=3). p values are indicated as: **** p < 0.0001, ** p < 0.01, and * p < 0.05. ns (not significant).

SIRP-ß2 was endogenously expressed in different myeloid effector cells, including PMNs, for which a large donor-dependent variability in expression was detected at the protein level (**Figure 1F**, **Figure 3A**). Notably, in mixed cultures of PMNs and cancer cell lines, PMN trogocytosis upon treatment with RTX yielded greatly varying levels of trogocytosis between donors. Specifically, from 10% to 45% for SUDHL-10 (**Figure 3B**), from 5% to 60% for SUDHL-6 (**Figure 3C**) and from 5% to 61% for Ramos (**Figure 3D**). Interestingly, a significant correlation between experimental trogocytosis (delta percentage between RTX and medium condition) and SIRP-ß2 expression was strongly detected for SUDHL-10 (R^2^ = 0.6363, p<0.001, **Figure 3E**) and SUDHL-6 (R^2^ = 0.7656, p<0.05 **Figure 3F**). Furthermore, after trogocytosis of tumor cells (SUDHL-10, SUDHL-6 and Ramos) the expression of SIRP-ß2 on PMNs was significantly decreased compared to medium control (**Figure 3G**). In contrast, the levels of SIRP-ß1 remained stable, whereas the expression of CD11b significantly increased (**Figures 3H, I**). Thus, SIRP-ß2 may play a role in anti-tumor immunity and was actively regulated during trogocytosis.

### 2.4 Interactome analyses of SIRP-ß2 identified DAP12 as signaling component and a strong association of SIRP-ß2 with MHC-class I

**Figure 4:**
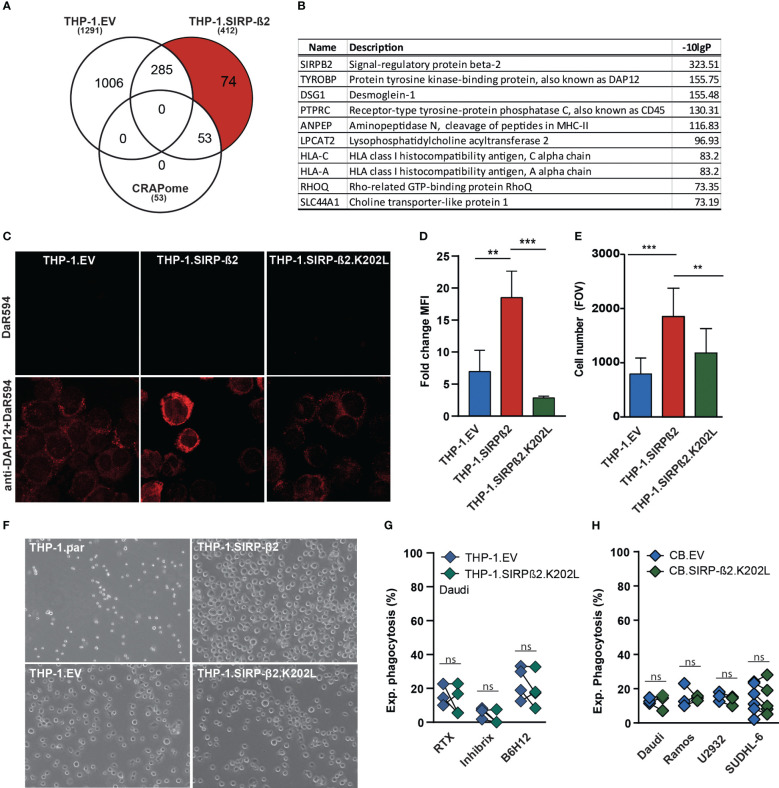
SIRP-ß2 with a point mutation in the charged lysine residue at position 202 (SIRP-ß2.K202L) in THP-1 cells did not augment adhesive and phagocytic effects. **(A)** Schematic GFP immunoprecipitation (IP), of THP-1.EV vs. THP-1.SIRP-ß2, including 127 unique protein hits of THP1-SIRP-ß2. **(B)** List of the 10 most frequently occurring proteins found in THP-1.SIRP-ß2, DAP12 as a leading hit. **(C)** Representative confocal microscopy images of anti-DAP12 staining on THP-1.EV, THP-1.SIRP-ß2 and THP-1.SIRP-ß2.K202L. **(D)** DAP12 expression on THP-1.EV, THP-1.SIRP-ß2 and THP-1.SIRP-ß2.K202L, determined by flow cytometry (n=3). **(E)** Quantitative adherent cells in field of view (FOV) of THP-1.EV, THP-1.SIRP-ß2 and THP-1.SIRP-ß2.K202L. **(F)** Representative images of adherent THP-1.parental, THP-1.EV, THP-1.SIRP-ß2 and THP-1.SIRP-ß2.K202L. **(G)** Experimental THP-1 mediated phagocytosis with Daudi cells for 3h, including RTX, Inhibrx and B6H12 treatment (1 µg/ml) (n=3). **(H)** Experimental CB-derived macrophage phagocytosis of EV vs. SIRP-ß2.K202L with Daudi, Ramos, U2932, SUDHL-6. p values are indicated as: *** p < 0.001 and ** p < 0.01. ns (not significant).

As demonstrated in macrophages and PMNs, SIRP-ß2 enhanced myeloid effector functions. To identify potential interaction partners of SIRP-ß2, SIRP-ß2 was immunoprecipitated (IP) followed by mass spectrometry (**Figure 4A**). Herewith, 127 unique interacting proteins were identified in THP-1.SIRP-ß2 compared to THP-1.EV, with 74 of these unique hits remaining upon exclusion of common contaminating background proteins, based on the CRAPome dataset (**Figure 4A**). The top 10 SIRP-ß2 co-IPed membrane proteins, based on -10logP, included both MHC complex proteins HLA-C and HLA-A and the ITAM-containing adaptor protein DAP12 or TYROBP (**Figure 4B**). DAP12 is involved in activation of NK cells, granulocytes, monocytes/macrophages and dendritic cells (22–24) and is required forSIRP-ß1 signaling (25, 26).

Specifically, a charged lysine in the transmembrane domain of SIRP-ß1 recruits DAP12. SIRP-ß2 contains a similarly charged lysine at amino acid position 202. To delineate whether DAP12 interaction was required for SIRP-ß2 signaling, lysine 202 was mutated to a non-charged leucine (K202L) and ectopically expressed in THP-1 (yielding THP-1.SIRP-ß2.K202L). Importantly, the introduction of the K202L mutation in SIRP-ß2 did not affect expression level or surface localization of SIRP-ß2 in THP-1.SIRP-ß2.K202L compared to THP-1.SIRP-ß2 (**Supplementary Figure 2A**). However, whereas fluorescent intensity of DAP12 staining was increased at the cell membrane of THP-1.SIRP-ß2 compared to EV, its intensity in THP-1.SIRP-ß2.K202L was comparable to THP-1.EV (**Figure 4C**). This finding was confirmed with flow cytometry, as DAP12 expression was significantly enhanced in THP-1.SIRP-ß2 compared to THP-1.SIRP-ß2.K202L or THP-1.EV (**Figure 4D**). At the same time, no changes in subcellular localization or expression level were detected for CD32, CD47 or SIRP-α for THP-1.SIRP-ß2.K202L, THP1.SIRP-ß2, and THP-1.EV (**Supplementary Figure 2B**). Interestingly, ectopic expression of SIRP-ß2 in the THP-1.SIRP-ß2.K202L cells did not augment adhesion of the cell line (**Figures 4E, F**) as seen in THP-1.SIRP-ß2, nor was phagocytosis of Daudi cells augmented (**Figure 4G**). Correspondingly, ectopic expression of SIRP-ß2.K202L in CB-derived macrophages did not enhance phagocytosis of Daudi, Ramos, U2932 or SUDHL-6 (**Figure 4H**). Thus, DAP12 is involved in the intracellular signaling pathway required for functional activity of SIRP-ß2.

**Figure 5:**
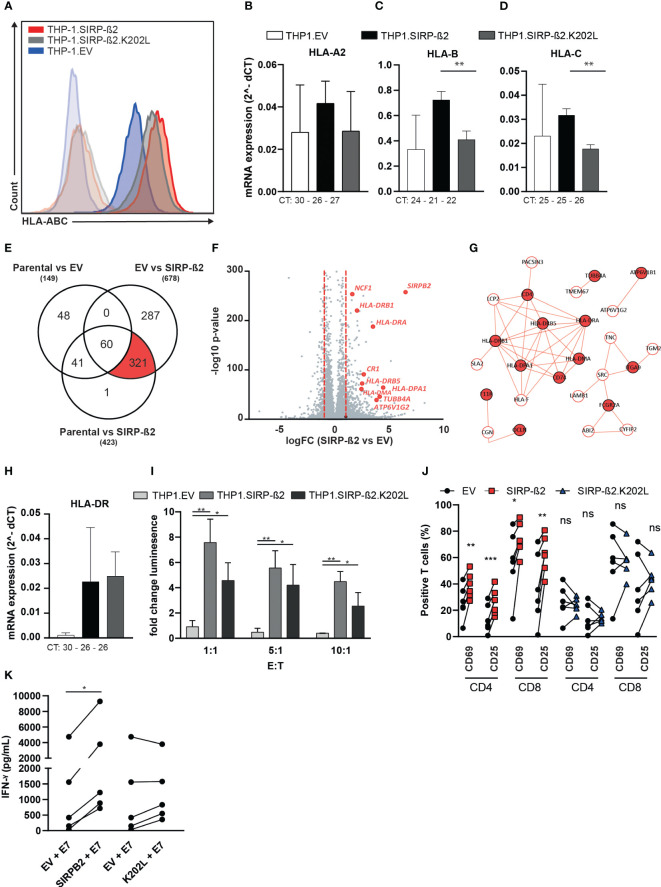
Interactome analyses of SIRP-ß2 identified DAP12 as signaling component and a strong association of SIRP-ß2 with MHC-class I and potentiate HPV-E7-specific T cell responses. **(A)** HLA-ABC expression of THP-1.EV, THP-1.SIRP-ß2 and THP-1.SIRP-ß2.K202L determined by flow cytometry. **(B)** mRNA expression of HLA-A2 (CT value 30 (EV), 26 (SIRP-ß2), 27 (SIRP-ß2.K202L)). **(C)** HLA-B (CT value 24 (EV), 21 (SIRP-ß2), 22 (SIRP-ß2.K202L)). **(D)** HLA-C (CT value 25 (EV), 25 (SIRP-ß2), 26 (SIRP-ß2.K202L)) n=3. **(E)** RNAseq analysis from THP-1.EV, THP-1.SIRP-ß2 and THP-1.SIRP-ß2.K202L, ~300 differently regulated genes. **(F, G)** Major upregulation of MHC-II from RNAseq analysis. **(H)** Quantitative mRNA HLA-DR expression on THP-1.EV, THP-1.SIRP-ß2 and THP-1.SIRP-ß2.K202L. **(I)** Activation of Jurkat Nuclear Factor of Activated T-cells (NFAT)-E7 with THP-1.EV, THP-1.SIRP-ß2 and THP-1.SIRP-ß2.K202L, including different Effector to Target (E:T) ratio’s. **(J)** Expression of CD69 and CD25 on CD4/CD8 T cells co-cultured with THP-1.EV, THP-1.SIRP-ß2 and THP-1.SIRP-ß2.K202L and E7 peptide for 6 h (n=4). **(K)** Quantitative IFN-γ secretion by primary E7-TCR specific T cells in co-culture with THP-1.EV, THP-1.SIRP-ß2 and THP-1.SIRP-ß2.K202L and E7 peptide for 48 h (n=5). p values are indicated as: *** p < 0.001, ** p < 0.01, and * p < 0.05. ns (not significant).

As described above, also MHC class I molecules potentially associated with SIRP-ß2 (**Figure 4B**). In subsequent flow cytometry analyses, HLA-ABC expression was found to be upregulated on THP-1.SIRP-ß2 (~2 fold) as well as on THP-1.SIRP-ß2.K202L (~1.4 fold) compared to EV control (**Figure 5A**). This overexpression was confirmed with an HLA-A2 specific antibody, specifically detecting the HLA-A2.01 allele expressed in THP-1 cells, yielding similar increases compared to THP-1.EV (THP-1.SIRP-ß2; 2.1 fold, THP-1.SIRP-ß2.K202L 1.4 fold, **Supplementary Figure 2C**). Notably, there were no significant differences in mRNA expression for HLA-A, HLA-B and HLA-C between THP-1.EV and THP-1.SIRP-ß2, suggesting there might be a transcriptionally independent upregulation of surface expression of MHC class I (**Figures 5B–D**). In line with this data, RNAseq analysis on THP-1, THP-1.EV and THP-1.SIRP-ß2, did not identify MHC class I as a differentially expressed cluster, although with the pool of ~300 differentially regulated genes (**Figure 5E**) a major upregulated gene cluster belonged to the MHC class II locus (**Figures 5F, G**). This differential mRNA expression of HLA-DR was confirmed by RT-qPCR (**Figure 5H**).

### 2.5 SIRP-ß2 expression potentiated HPV-E7-specific T cell responses

Since MHC molecules are directly responsible for antigen presentation, expression of SIRP-ß2 and subsequent upregulation of MHC molecules might potentiate antigen presentation and increase activation of specific T cell responses. To test this hypothesis, a Jurkat.NFAT-luciferase reporter assay with a Human Papilloma Virus (HPV) E7 peptide specific TCR, yielding luminescence upon TCR triggering, was utilized to investigate TCR signaling (27). Upon E7 peptide pulsing, the THP-1.SIRP-ß2 as well as the SIRP-ß2.K202L cells significantly increased NFAT activation compared to THP-1.EV at Effector-to-Target (E:T) ratios of 1:1, 5:1 and 10:1 (**Figure 5I**). When primary E7-TCR specific T cells were co-cultured with THP-1.SIRP-ß2 and E7 peptide, a significant increase in CD69 and CD25 was observed in both CD8 and CD4 positive T cells of different donors compared to co-cultures with THP-1.EV and E7 peptide (**Figure 5J**). In these primary T cells the co-culture with THP-1.SIRP-ß2.K202L did not enhance T cell activation (CD69 or CD25) compared to THP-1.EV (**Figure 5J**). Finally, IFN-γ secretion by the primary E7-TCR specific T cells was E7 dependent (**Supplementary Figure 2D**) and significantly increased only in co-cultures with THP-1.SIRP-ß2 compared to THP-1.EV (p< 0.05, **Figure 5K**). In conclusion, SIRP-ß2 might enhance antigen presentation by upregulation of MHC-I class and increase T cell activation.

## 3 Discussion

In the current study, we identified that SIRP-ß2 is expressed under normal physiological conditions in macrophages and granulocytes at the mRNA and protein level and that endogenous expression of SIRP-ß2 on PMNs correlated with trogocytosis of cancer cells. Furthermore, ectopic expression of SIRP-ß2 in the THP-1 monocytic cell line and in primary cord blood-derived macrophages increased adhesion, differentiation, and cancer cell phagocytosis. SIRP-ß2 recruited the immune activating adaptor protein DAP12 to positively regulate innate immunity, with a mutation of the charged lysine responsible for DAP12 interaction abrogating functional activity. Finally, ectopic expression of SIRP-ß2 on the THP-1 model enhanced surface expression of MHC-I molecules, enhanced T cell activation as seen by increased NFAT activation in a Jurkat report system and the upregulation of activation markers CD69 and CD25 and IFN-γ secretion on primary T cells. These findings position SIRP-ß2 as a novel positive regulator of innate immunotherapy.

SIRP-ß2 was first documented by Ichigotani et al., who isolated full-length cDNA encoding SIRP-ß2 (18). SIRP-ß2 has high homology with SIRP-ß1, with 75.5% of the amino acid sequence being identical. The main difference is in the extracellular domain, in which SIRP-ß1 has one V- and two C1-set Ig domains, whereas SIRP-ß2 contains two V-set Ig domains (2). Both proteins lack any tyrosine-phosphorylation sites, but contain a single basic lysine residue within the hydrophobic transmembrane domain. For SIRP-ß1, this charged residue was shown to associate with the adaptor protein DAP12, which through its cytoplasmic single ITAM can induce activating pathways, such as increased adhesion and phagocytosis (25, 28, 29). Therefore, DAP12-mediated activation was also the proposed mechanism for SIRP-ß2 (2, 18). The results presented here confirm this hypothesis, with SIRP-ß2 signaling being clearly dependent on DAP12 recruitment. Indeed, a mutation of the charged lysine residue on SIRP-ß2 lead to a complete loss of the increased adhesion and phagocytosis observed in THP-1.SIRP-ß2 and CB.SIRP-ß2 cells. In addition, DAP12 was found to be associated with SIRP-ß2 (IP), in SIRP-ß2 but not EV THP-1 cells. Finally, protein expression of DAP12 was increased in THP-1.SIRP-ß2 but not SIRP-ß2.K202L or EV cells. Together, this indicates that SIRP-ß2 signaling is mediated through DAP12, with the DAP12-SIRP-ß2 interaction facilitated by a single lysine residue in the transmembrane region. Interestingly, the HPV16-EV TCR Jurkat.NFAT.luciferase model in **Figure 5I** showed a significant difference between THP-1.EV and THP-1.SIRP-ß2.K202L, whereas in the primary T cell model these differences were not found (**Figure 5J**). However, in here we are looking at two different read-out experiments: NFκB signaling in a THP-1 model and surface expression of CD69 and CD25 on primary T cells. Primary T cells experiments are more sensitive to activation stimuli, also to time (best window for CD25 and CD69 can vary over time), different T cell populations and donor variability. The EV control in primary T cells express already high levels of activation markers, so this might be the reason why we don’t see the window with the SIRP-ß2.K202L variant. Also, we have previously encountered a similar difference between the Jurkat.NFAT.luciferase model system and primary cell type (27).

Whereas the immune-inhibitory receptor SIRP-α as well as the T cell expressed SIRP-γ both bind to CD47, SIRP-ß2 did not bind to CD47 (**Supplementary Figure 2E, F**). Interestingly, DAP12 signaling required (antibody-mediated) crosslinking of SIRP-ß1, which has also been reported for other ITAM containing adaptor proteins (17, 25, 28–30). However, SIRP-ß2 appeared to be active without secondary cross-linking, which could indicate that SIRP-ß2 does not require a ligand to operate or has the ability to self-cluster. Alternatively, the ligand was present in either the medium or expressed intracellularly. The latter seems unlikely, as immunoprecipitation did not reveal any suitable ligand candidates. Notably, whereas SIRP-ß2 already activates signaling in the current assays, it would be of interest to investigate whether monoclonal agonistic crosslinking of SIRP-ß2 could further enhance activity. In this respect, SIRP-ß1 has recently been exploited as immunotherapeutic target, with targeting of SIRP-ß1 on macrophages promoting phagocytic activity, promoting polarization towards M1 phenotype, and inducing killing of murine bladder cells (31). If SIRP-ß2 responds to agonistic targeting, it could be exploited as a co-stimulatory therapeutic target of the innate immune system.

Endogenous expression of SIRP-ß2 on the protein level was only found in a subset (~45% **Figure 1F**) of donors on myeloid cells and, interestingly, was rapidly down regulated during trogocytosis in PMNs of different donors. Trogocytosis was previously reported to require active signaling via Fc-γreceptors that have bound opsonizing antibodies, required CD11b/CD18 integrin and was inhibited by CD47/SIRP-α signaling (32, 33). Notably, for SIRP-ß2, but not SIRP-ß1, a clear correlation was found between endogenous expression levels expression levels on PMNs and the level of trogocytosis. Together with the active down-regulation during trogocytosis, this positions SIRP-ß2 as a stimulatory receptor modulating trogocytosis. The mechanisms behind the downregulation of SIRP-ß2 during trogocytosis requires further investigation, but might be due to internalization or to shedding of the extracellular domain. In general, ectodomain shedding plays an important role in various processes, such as, migration, adhesion and different immune responses. Such shedding has been reported previously for SIRP-α (34, 35). Neural activity is involved in synapse maturation, which is activity-dependent on the ectodomain shedding of SIRP-α (36). The reason of shedding of the extracellular domain of SIRP-α is thought to be the enhanced binding capacity to the presynaptic receptor CD47 (36). In addition, the shedding of ectodomain SIRP-α in THP-1 monocytes and lung epithelia cells was regulated by metalloproteinase domain-containing protein 10 (ADAM10). In here, the shedding enhanced immune activation signaling in response to inflammatory signaling (37).

Interestingly, whereas HLA-A2 was upregulated on the protein level this was not mirrored by mRNA levels, which were comparable for SIRP-ß2, SIRP-ß2.K202L and EV THP-1 cells. Thus, the increased HLA expression is not due to transcriptional upregulation. MHC-I complexes are dynamic molecules that undergo dynamic endocytic cycling and turnover (38, 39). Only small amounts are normally exported to the cell surface, while most class I molecules are retained in the ER. Furthermore, once at the cell surface MHC class I molecules are continually removed by endocytosis and either recycled or degraded (40). We hypothesize that SIRP-ß2 influences the surface stability of MHC class I molecules or reduces degradation via direct interaction with the complex, as IP showed that both HLA-A and HLA-C potentially associated with SIRP-ß2. A reverse IP targeting HLA-A and HLA-C could provide more evidence.

In conclusion, SIRP-ß2 is expressed on myeloid cells at protein level and enhanced adhesion, differentiation, cancer cell phagocytosis and T cell activation in an *in vitro* setting. DAP12 is responsible for SIRP-ß2 signaling, and is recruited through a single, positive lysine residue in the transmembrane domain of SIRP-ß2. Thus, SIRP-ß2 appears to be a positive regulator of innate anticancer immunity.

## 4 Methods

### 4.1 Cell lines, peripheral blood mononuclear cells and primary normal hematopoietic cells

The cell lines THP-1, HL-60, Daudi, Ramos, U2932, DLD-1, OVCAR-3, SUDHL-6, SUDHL-10, and Jurkat were obtained from the American Type Culture Collection (ATCC, Manassas, VA). The Jurkat.NFAT.luciferase reporter cell line was obtained from BPS Bioscience (San Diego, CA). All cell lines were cultured at 37 °C in a humidified 5% CO_2_ containing atmosphere. SUDHL-10 was cultured in RPMI 1640 medium (Lonza, Basel, Switzerland) supplemented with 20% fetal calf serum (FCS)(Thermo Scientific Waltham, MA, USA). The rest of the cell lines were cultured in RPMI 1640 medium supplemented with 10% fetal calf serum. Polymorphonuclear neutrophils (PMNs) and monocytes were isolated from peripheral blood mononuclear cells (PBMCs) from healthy donors using ficoll density gradient (Lymphoprep™, Bernburg, Germany). For PMNs; The pellet after lymphoprep was resuspended in 1X red blood cell lysis (Sigma Aldrich, St. Louis, MO, USA) for 15 min at RT. Whereafter it was washed twice with PBS and resuspended in RPMI 1640 supplemented with 10% FCS. From the mononuclear fraction, monocytes were isolated using CD14 microbeads following the manufactures protocol (Miltenyi Biotec, Leiden, The Netherlands).

Umbilical cord blood (CB) was derived from healthy full-term pregnancies after informed consent from the Obstetrics department of the Martini Hospital and the University Medical Center Groningen (UMCG), the Netherlands (protocol code NL43844.042.13, 6 January 2014). Mononuclear cells (MNCs) were isolated by density gradient centrifugation using lymphoprep (Alere Technologies AS, Oslo, Norway) and CD34+ cells were selected using the MACS CD34 microbeads kit on autoMACS (Miltenyi Biotec, Leiden, The Netherlands). Purity of >96% CD34+ cells after isolation was confirmed using Cytoflex (Beckman Coulter, Brea, CA, USA).

### 4.2 Lentiviral production

EV, SIRP-ß2 short and long isoform, SIRP-ß2.K202L were cloned in the pRRL vector, containing GFP separated by an IRES sequence. On day 1, T75 cultures flasks were coated for 2 h with 0.1% gelatin, afterwards 3x10^6^ HEK293T cells were plated in 10 mL DMEM (Lonza, Basel, Switzerland) supplemented with 10% FCS. After overnight culture, the HEK293T cells were transfected with a mix of 3 µg packaging construct, 0.7 µg glycoprotein envelop plasmid, 3 µg vector construct and FuGENE HD transfection reagent (FuGENE^®^ HD transfection Reagent, Promega, Madison, WI, USA) was added in a DNA construct:FuGENE ratio of 1:7, by dropwise addition. After 18 h medium was replaced with DMEM medium without FCS, and incubated for 24 h. Afterwards, the virus containing supernatant was collected, spun down at 450 g for 5 min and passed over a 0.45 µM filter using a syringe. THP-1 and HL-60 cells were plated 5x10^5^/ml in complete medium in a 6-wells plate. 100 µl of virus was added to the cells, after 24 h of incubation, virus was washed 3x with PBS, supplemented with 2% FCS, by spinning down at 450 g for 5 min.

### 4.3 *In vitro* THP-1 phagocytosis assay

THP-1 transduced with GFP expressing vector, SIRP-ß2 and SIRP-ß2.K202L cells were sorted with the cell sorter (Sony SH800, FACS facility, UMCG) and cultured in complete medium. THP-1 cells were seeded at 1.0x10^4^ cells/well in a 24-well plate and incubated with 1 µM PMA (Thermo Scientific Waltham, MA, USA), for 72 h. After 72 h, PMA was washed away from the THP-1 cells. Tumor cells were labeled with Incucyte Cytolight Red (Essen BioScience, Ann Arbor, MI, USA) according to manufacturer’s instructions. The labeled tumor cells were added to the THP-1 like macrophages at an effector to target ratio of 1:2, after which anti-CD20 or anti-CD47 antibody (1 µg/mL) was added and incubated for 3 h at 37°C. Subsequently, tumor cells were gently removed from the THP-1 like macrophages by washing 2-3 times with PBS and THP-1 macrophages were detached from the plate using TrypLE (Thermo Fisher Scientific, Waltham, MA, USA). Phagocytosis was analyzed by taking fluorescent pictures of the phagocytosis plate by use of the Incucyte microscope (S3 Live-Cell Analysis System, Ann Arbor, MI, USA) or determined by flow cytometry. The percentage of THP-1 mediated phagocytosis was calculated by counting the number of macrophages containing red tumor cells inside per 100 macrophages. Each condition was quantified by evaluating three randomly chosen fields of view. For flow cytometry analysis, the percentage of phagocytosis was determined by quantifying the percentage of double-positive (GFP+/incucyteRed+) THP-1 cells.

### 4.4 *In vitro* CB derived macrophage phagocytosis assay

Freshly isolated CD34+ cells from CB were first expanded in Stem Line medium (Miltenyi Biotech, Leiden, The Netherlands) supplemented with: 100 ng/mL SCF (Sigma Aldrich, St. Louis, MO, USA), 50 ng/mL FLT-3 (Sigma Aldrich, St. Louis, MO, USA), 30 ng/mL GM-CSF (ImmunoTools, Friesoythe, Germany) and 10 ng/mL IL-6 (Sigma Aldrich, St. Louis, MO, USA). After expansion, these cells were transduced with GFP expressing vector, SIRP-ß2 short, long isoform and SIRP-ß2.K202L. To differentiate the CD34+ towards macrophages, cells were cultured in RPMI 1640 supplemented with 20% FCS and 50 ng/mL M-CSF (ImmunoTools, Friesoythe, Germany) for 14 days. Hereafter, macrophages were polarized into M1 like macrophages using 20 ng/mL IFNy and 50 ng/mL LPS (ImmunoTools, Friesoythe, Germany) for 24 h. For CB-derived macrophage phagocytosis assay, macrophages were detached by TrypLE and pre-seeded at 1.0x10^4^ cells/well in a 96-well plate. Tumor cells were labeled with Incucyte Cytolight Red according to manufacturer’s instructions. Labeled tumor cells were mixed with the macrophages at an effector to target (E:T) ratio of 1:5 and treated with anti-CD20 (1 µg/mL) for 3 h at 37°C. Tumor cells were gently removed from the macrophages by washing 2-3 times with PBS. Phagocytosis was analyzed by taking fluorescent pictures using the IncuCyte S3 Live-Cell Analysis System. The percentage of phagocytosis was calculated by counting the number of macrophages containing red tumor cells inside per 100 macrophages. Each condition was quantified by evaluating three randomly chosen fields of view. Experimental phagocytosis was calculated using phagocytosis with anti-CD20 subtracted by the phagocytosis of the medium control.

### 4.5 Confocal microscopy

To detect endogenous SIRP-ß2 expression on immune cells, such as monocytes, macrophages and granulocytes, 5 x 10^4^ cells were transferred to a microscopic slide using a cytocentrifuge (Cytospin 3, Shandon, England). Cells were fixed with 4% w/v paraformaldehyde (PFA) for 15 min and subsequently blocked with PBS supplemented with 5% Horse Serum (HS) (Sigma Aldrich, St. Louis, MO, USA) for 45 min at RT. Cells were incubated with SIRP-ß2 antibody (#P61637, Thermo Fisher Scientific, Waltham, MA, USA) for 1 h incubation at RT, washed 3 times with PBS-Tween20 (0.01%) (Sigma Aldrich, St. Louis, MO, USA), followed by incubation with Goat-anti-Rabbit-488 (#A-11034, Thermo Fisher Scientific, Waltham, MA, USA) and CD11b-APC (#101212 ImmunoTools, Friesoythe, Germany) for 30 minutes at RT. Cells were then washed 3 times with PBS-Tween20 (0.01%) and treated with mounting medium containing DAPI (Sigma Aldrich, St. Louis, MO, USA). Representative images were taken using a Leica SP8 Confocal microscope (Leica Microsystems, Rijswijk, The Netherlands). The same procedure was followed to detect DAP12 (130-155-088, Miltenyi Biotec, Leiden, The Netherlands) on THP-1.EV, THP-1.SIRP-ß2 and THP-1.SIRP-ß2.K202L.

### 4.6 RNA expression

Expression across the hierarchical differentiation tree of the hematopoietic system was investigated using BloodSpot (21) for SIRP-ß2, SIRP-α and SIRP-ß1 of the SIRP family, using BloodSpot. To determine mRNA levels of MHCI/II related genes (HLA-A2, HLA-B, HLA-C and HLA-DR) as well as mRNA levels of SIRP-α, SIRP-ß1, SIRP-ß2, SIRP-γ and SIRP-δ, on monocytes and T cells, 5x10^5^ monocytes, T cells or THP-1 cells expressing EV, SIRP-ß2 or SIRP-ß2.K202L were harvested into an Eppendorf tube and washed in PBS. Subsequently, the cell pellet was resuspended in 350 µL RLT buffer of the mRNA isolation kit (Qiagen RNeasy plus mini kit #74134). Afterwards mRNA was isolated following the manufacturers protocol. cDNA was synthesized using an iScript™ cDNA Synthesis Kit (Biorad; #1708891), following manufacturer protocol for 1000 ng/20 μL. 5 μL SYBRgreen (Biorad, #1725274) was added to 5 ng cDNA per condition. The RT-qPCR program on the thermocycler (Biorad C1000, CFX384 Real Time System) consisted of: 3 min 95°C; (5 s 95°C, 15 s 58°C → 39 times); 3 s 65°C; 5 s 95°C, and used the following primer sequences (5’- 3’):

HLA-A2; Fwd: *TCCTGCTACTCTCGGGGGCT*, Rev: *CTCCCACTTGTGCTTGGTGG*. HLA-B; Fwd: *CTGCTGTGATGTGTAGGAGGAAG*, Rev: *GCTGTGAGAGACACATCAGAGC*. HLA-C; Fwd: *GGAGACACAGAAGTACAAGCGC*, Rev: *ACATCCTCTGGAGGGTGTGAGA*. HLA-DR; Fwd: *AGAGACAGTCTTCCTGCCCA*, Rev: *TGGAGCATCAAACTCCCAGTG*. SIRP-alpha; Fwd: *TCTACAAGGTTGCATGAG*, Rev: *GGTTCAGGTCTGCATATGTG*. SIRPß1; Fwd: *GAATTCATGCCCGTGCCAGC*, Rev: *GAATTCGGAGGAGGCAGCAGAG*. SIRPß2; Fwd: *CAGCCTGAAAGTGAAAGCA*, Rev: *AGGTTCACTGGTGAATTCTG*. SIRPγ; Fwd: *AGATCTATGCCTGTCCCAGCC*, Rev: *GAATTCGGGAGGAAGGAGAG*. SIRP-δ; Fwd: *AGGCATTCAGGCAGAGCAAG*, Rev: *CCAGTTCAAGCAGCAGATACAG*.

RT-qPCR data was analyzed as followed: first deltaCT was calculated using the CT-values of gene of interested minus housekeeping gene (RPL27), whereafter the calculation of 2^-deltaCT was made.

### 4.7 Adhesion assay

To determine adhesion of THP-1.EV, THP-1.SIRP-ß2 and THP-1.SIRP-ß2.K202L, 7.5x10^5^ cells were added into a 6-well plate and incubated for 4 or 24 h in the presence or absence of 2.5 µg/mL PMA. After incubation, the wells were gently washed twice with PBS and stained with crystal violet (Sigma Aldrich, St. Louis, MO, USA) (1:3 dilution) for 1 h at RT. The crystal violet staining buffer was removed and wells were washed six times with PBS. Photos were taken on day 2, 4 and 7 to screen for adhesion, using an auto screen machine (AID EliSpot reader). Adhesion assay using xCELLigence, was performed as described previously (41). In short, 96-well E-plates (E-plate 96) (ACEA Bio, catalog number: 5232368001) were coated with extracellular matrix (ECM) molecule fibronectin (bovine plasma) (Sigma-Aldrich, catalog number: 341631) for 1 h at 37°C, whereafter excess coating was removed by washing twice with PBS. To block the non-specific binding of the THP-1 and HL-60 cells, the plate was coated for 1h at 37°C with 100 µl 0.1% BSA. For the experiment, 3x10^4^ cells were plated in an end volume of 150 µl complete medium and measured for 4h in real time.

### 4.8 Trogocytosis

PMNs were isolated from apheresis blood of healthy individuals using ficoll density gradient using lymphoprep, as described above. SIRP-ß2 antibody was used to detect the expression level of endogenous SIRP-ß2 on PMNs. After 1 h of incubation with SIRP-ß2 antibody on ice, cells were washed thrice with PBS and incubated with Goat-anti-Rabbit-488 for 30 minutes on ice. Cells were washed thrice with PBS and analyzed using flow cytometry.

For trogocytosis by PMNs, SUDHL-6, SUDHL-10 and Ramos cells were labeled with Incucyte Cytolight Red according to manufacturer’s instructions and mixed with PMNs in a 1:1 ratio (5x10^4^: 5x10^4^) with/without anti-CD20 antibody (1 µg/mL) for 3 h at 37°C. Analysis was performed using flow cytometry. For analysis, the percentage of trogocytosis was determined using incucyteRed+ tumor cells within the PMN gate. Experimental trogocytosis (trogocytosis in presence of RTX subtracted by trogocytosis medium control).

### 4.9 DAP12 intracellular staining

5x10^5^ THP-1 cells expressing EV, SIRP-ß2 or SIRP-ß2.K202L were fixed and permeabilized according to manufacturer’s protocol (fix and perm kit, Thermo Scientific Waltham, MA, USA). During permeabilization, isotype control or DAP12 antibody (130-155-088, Miltenyi Biotec, Leiden, The Netherlands) was added to the cells during the permeabilization step. Cells were then washed thrice in PBS containing 2% BSA and 0.5 mM EDTA, before analysis using flow cytometry. DAP12 expression was calculated as the fold increase in Mean Fluorescent Intensity (MFI) between isotype and DAP12 stain (MFI DAP12 stain/MFI isotype stain). Data is represented as mean ± standard deviation of three (THP-1.SIRP-ß2.K202L) or four (THP-1.EV, THP-1.SIRP-ß2) independent experiments. Statistical analysis was performed in GraphPad Prism software (GraphPad Prism V9.1.0; GraphPad Software, La Jolla, CA, USA) by a paired one-way ANOVA with Geisser-Greenhouse correction followed by a Tukey-test.

The Jurkat activation assay was performed as described in (27). In brief, THP-1.EV, THP-1.SIRP-ß2 and THP-1.SIRP-ß2.K202L were plated in a 96 well plate at 1x10^4^ cells per well, followed by a 2 h incubation (37°C, 5% CO_2_) with or without 10 µg/mL of E7 HPV peptide (E711-20, Peptides & Elephants, Berlin, Germany). Subsequently, Jurkat.NFAT.LucE7 or Jurkat.NFAT.Luc were added to THP-1 cells at 10:1, 5:1 and 1:1 E:T ratios, whereupon the mixed culture was incubated for 6 h (37°C, 5% CO_2_). Luminescence was quantified by transfer of 100 µl suspension to a 96-well white plate (CLS3922-100EA, Merck, Darmstadt, Germany) to which 30 µL of Bio-Glo™ (G7941, Promega, WI, USA) was added. After 15 min, luminescence was determined using a luminescence reader at 37°C (Synergy, BioTek, Vermont, USA). Data was acquired using the Gen5 (V2.03) software with the following settings: (emission:hole, optics:top, gain:auto, integration time:1 sec, read height: 1 mm). The fold change in luminescence was calculated as follows:


$$Fold\;change=\frac{\left[sample\;luminescence\right]-\left[control\;luminescence\right]}{\left[control\;luminescence\right]}$$

With control luminescence defined as Jurkat.NFAT.LucE7 cells treated without HPV E7 peptide.

### 4.10 Primary T cell activation

Primary T cells were obtained by isolation of PBMCs from buffy coats of HLA-A2 negative donors by ficoll density gradient lymphoprep, followed by pan-T cell isolation according to manufacturer’s protocol (139-096-535, Miltenyi Biotec, Leiden, The Netherlands). T cells were lentivirally transduced with EV or TCR-E7 as described in (27). THP-1.EV, THP-1.SIRP-ß2 and THP-1.SIRP-ß2.K202L were inactivated for 1 h (37°C) by incubation with 6 µg/mL Mitomycin-C, followed by washing thrice in PBS. 100 µL of inactivated cells were plated at 1x10^4^ cells per well, followed by a 2 h incubation (37°C, 5% CO_2_) with or without 10 µg/mL of E7 HPV peptide (E711-20, Peptides & Elephants, Berlin, Germany). Then, 100 µL of T cells (expressing EV or TCR-E7) were added to the THP-1 cells at a 5:1 E:T ratio (based on GFP positive T cells) and incubated for 48 h (37°C, 5% CO_2_). Samples were collected, spun down (300 xG, 5 min) and stained for CD3-BV785 (317330, BioLegend, San Diego, USA), CD8-BV421 (344748, BioLegend, San Diego, USA), CD69-PerCP (310928, BioLegend, San Diego, USA) and CD25-APC (21810256, ImmunoTools, Friesoythe, Germany) and analyzed using flow cytometry. The supernatant was analyzed for IFN-y, using an IFN-y cartridge (SPCKB-PS-002574, Biotechne, Minneapolis, USA) with the Ella automated immunoassay system, following manufacturer’s protocol.

### 4.11 Colony formation unit assay

The Colony Formation Unit (CFU) assay was performed in methylcellulose supplemented with 20% IMDM, 1% penicillin-streptomycin (Thermo Scientific Waltham, MA, USA) and 20 ng/mL IL-3 (Sigma Aldrich, St. Louis, MO, USA), IL-6 (Sigma Aldrich, St. Louis, MO, USA), SCF (Sigma Aldrich, St. Louis, MO, USA), G-CSF (ImmunoTools, Friesoythe, Germany), and 1 U/mL erythropoietin (Sigma Aldrich, St. Louis, MO, USA). Freshly isolated CD34+ cells derived from cord blood (CB) were transduced with GFP expressing vector (EV), SIRP-ß2 and SIRP-ß2.K202L. 1x10^3^ cells were sorted with the MoFlo Astrios cell sorter (Beckman Coulter, Brea, CA, USA) into a Petri dish and incubated (37°C, 5% CO_2_). After 14 days the cultures were scored based on colony formation.

### 4.12 GFP-immunoprecipitation

2.5x10^3^ µg total protein was lysed in lysis buffer (150 mM NaCl, 50 mM Tris, pH 7.2, 20 nM EDTA, 20 nM EGTA, 1% NP-40 (12879500, Roche, Basel, Switzerland), 0.1% SDS (L6026, Sigma Aldrich, St. Louis, MO, USA); containing Na_3_CO_4_ (450243, Sigma Aldrich, St. Louis, MO, USA) and protease inhibitor cocktail (Sigmafast; S8820, Sigma Aldrich, St. Louis, MO, USA), and protein concentration was determined using a Bradford assay (Pierce™ Coomassie (Bradford) Protein Assay Kit, #23200, Thermo Scientific, Waltham, MA, USA). Pre-clearance beads (25 μL per sample, Chromotek bmab-20) were washed thrice with ice cold wash buffer (10 mM Tris, 150 mM NaCl, 0.5 mM EDTA), and subsequently added to the lysate (2,5 x10^3^ μg total protein in 400 μL lysis buffer). After 1 h incubation at 4°C (on end-over-end tumbler), the lysate was harvested in a Eppendorf tube and added to pre-washed GFP-beads (25 μL per sample, Chromotek gtma-20). After 1 h incubation at 4°C (end-over-end tumbler), the supernatant was removed and beads were washed thrice with ice cold wash buffer. Subsequently, beads were resuspended in 100 μL SDS-sample buffer (Laemmli buffer) and boiled for 10 minutes. The supernatant was harvested using a magnetic field and 40 μL was used for analysis with mass spectrometry (Orbitrap-LC-MS).

Accession numbers and -10logP values were used for further data processing. First, the acquired accession numbers of THP-1.EV and THP-1.SIRP-ß2 mass spectrometry were compared and only unique SIRP-ß2 accession numbers were kept. Next, all unique hits with an occurrence of 20% or more in the CRAPome database (42) were filtered out. CRAPome is a large publicly available database of standardized negative controls, obtained from various leading lab specializing in affinity purification mass spectrometry. The cellular location of the remaining proteins was determined, using the accession numbers and the UniProt database. Lastly, only the membrane-bound proteins were selected and ranked based on -10logP values.

### 4.13 RNA sequencing

RNA was isolated from THP.1-EV and THP.1-SIRP-ß2 using RNA isolation columns (Qiagen RNeasy plus mini kit #74134). 0.1 µg RNA was send for sequencing to BGI (BGI Group Guangdong ICP 10059378) and analyzed following protocol of the company. Library type: DNBSEQ Eukaryotic Strand-specific mRNA library Sequencing Platform: DNBseq Sequencing read Length: PE150 Clean fastq phred quality score encoding:Phred+33.

### 4.14 THP-1 phenotyping by flow cytometry

5x10^5^ THP-1 cells expressing EV, SIRP-ß2 or SIRP-ß2.K202L spun down (300 xG, 5 min) and resuspended in PBS containing 2% BSA and 0.5 mM EDTA. Then isotype control, HLA-ABC (21159036, ImmunoTools, Friesoythe, Germany), HLA-A2 (17-9876-42, Invitrogen), CD32-PE (303205, BioLegend), CD47-APC (323124, BioLegend), or CD172a-APC (372106, BioLegend) were added and incubated for 30 min at 4°C Celsius. Then, the cells were washed three times in PBS containing 2% BSA and 0.5 mM EDTA, before analysis using flow cytometry. HLA-ABC and HLA-A2 fold increase was calculated as the MFI fold increase between isotype and staining (MFI stain/MFI isotype stain). The fold change was normalized to THP-1.EV.

### 4.15 Statistical analysis

Statistical significance was determined using a Student’s t-test or paired one-way ANOVA with Geisser-Greenhouse correction followed by a Tukey-test, using Graphpad Prism software (GraphPad Prism V9.1.0; GraphPad Software, La Jolla, CA, USA). p values are indicated as: **** p< 0.0001, *** p< 0.001, ** p< 0.01, and * p< 0.05.

## Data availability statement

The original contributions presented in the study are publicly available. This data has been deposited to the ProteomeXchange Consortium via the PRIDE partner repository with the dataset identifier: PPXD047166.

## Ethics statement

The studies involving humans were approved by protocol code NL43844.042.13, 6 January 2014. The studies were conducted in accordance with the local legislation and institutional requirements. The participants provided their written informed consent to participate in this study.

## Author contributions

NV: Data curation, Formal Analysis, Investigation, Methodology, Writing – original draft, Writing – review & editing. LN: Formal Analysis, Investigation, Methodology, Writing – original draft, Writing – review & editing. YH: Formal Analysis, Investigation, Methodology, Writing – review & editing. HL: Formal Analysis, Investigation, Software, Writing – review & editing. MC: Investigation, Writing – review & editing. GH: Conceptualization, Resources, Writing – review & editing. VW: Formal Analysis, Investigation, Methodology, Writing – review & editing. JS: Conceptualization, Resources, Supervision, Writing – review & editing. EB: Conceptualization, Funding acquisition, Methodology, Project administration, Resources, Supervision, Writing – original draft, Writing – review & editing.
