# Supplementary material for: Signal regulatory protein beta 2 is a novel positive regulator of innate anticancer immunity
Source: Front Immunol. 2023 Dec 5;14:1287256. doi: 10.3389/fimmu.2023.1287256 (PMC10729450; doi:10.3389/fimmu.2023.1287256)
Supplement: Supplementary file 1 [file DataSheet_1.pdf]

Supplementary Material

Signal Regulatory Protein Beta 2 is a novel positive regulator of innate anticancer immunity

Nienke Visser, Levi Collin Nelemans, Yuan He, Harm Jan Lourens, Macarena González Corrales, Gerwin Huls, Valerie R. Wiersma, Jan Jacob Schuringa, Edwin Bremer[#]

Author affiliations

Department of Hematology, University of Groningen, University Medical Center Groningen (UMCG), Groningen, The Netherlands

Key words: SIRP-ß2; CD47; phagocytosis; macrophage; antigen presentation

[#]Correspondence to:

Prof. Dr. E Bremer, University Medical Center Groningen (UMCG), Groningen 9713 EZ, The Netherlands, e.bremer@umcg.nl

Suppl. Fig. 1

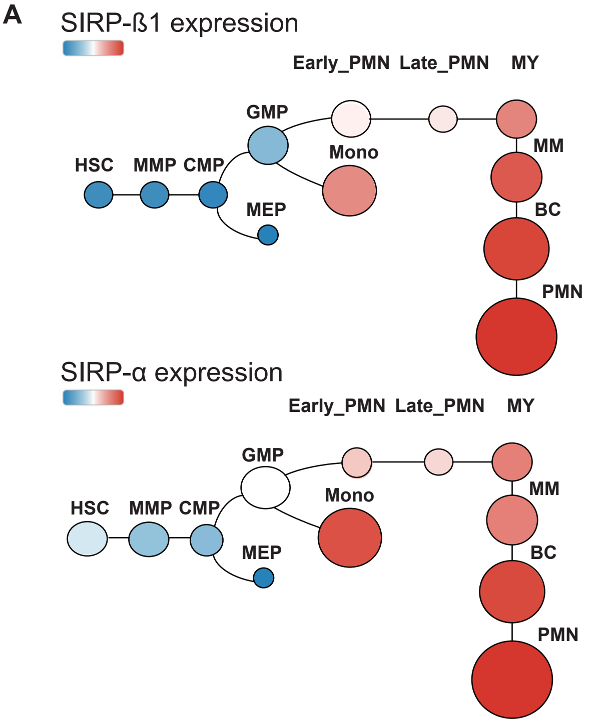

B

	Amount of counted colonies (n=3)								
	NT			EV			SIRP-ß2		
CFU-M	68	54	43	25	30	42	52	34	30
CFU-G	32	34	43	25	52	36	35	46	22
CFU-GM	22	5	9	6	8	8	5	1	18
BFU-E	32	68	74	40	38	30	18	34	28

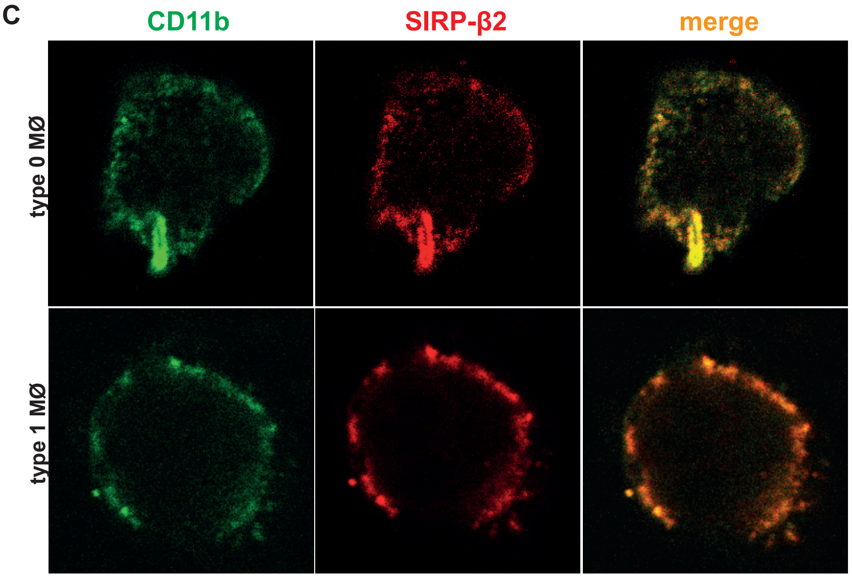

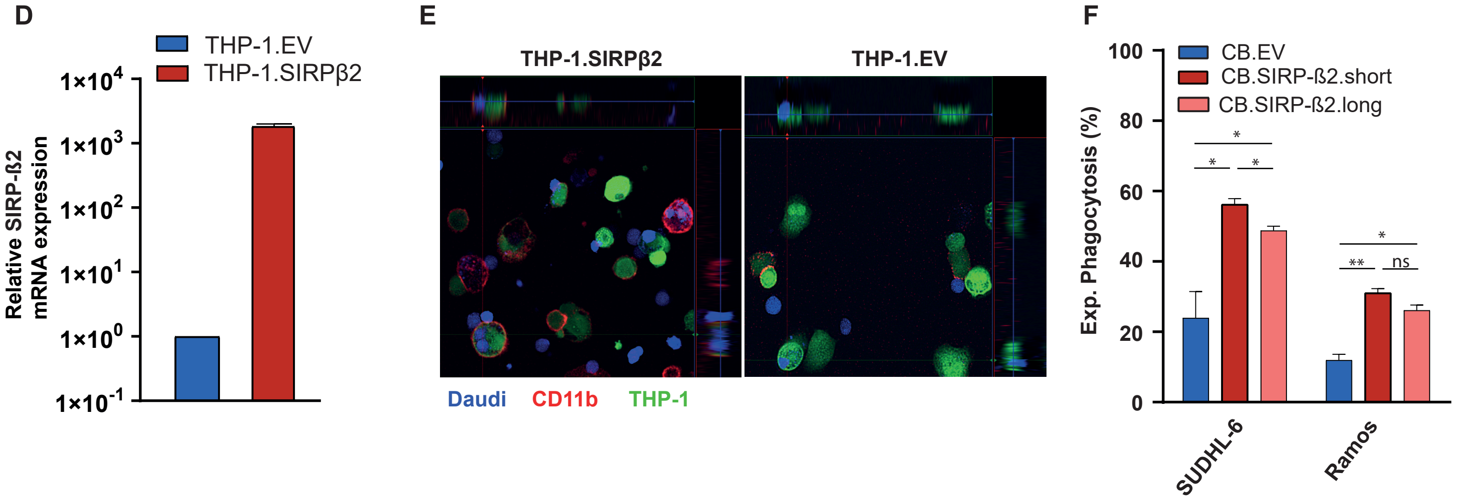

Suppl. Fig 1. Expression pattern and functional phagocytosis activity of SIRP-ß2 **(A)** mRNA SIRP-ß1 (above) and SIRP-α (below) expression in hierarchical differentiation tree. **(B)** Original data derived from counted colonies of the Colony Formation Unit assay after 14days, NT, EV and SIRP-ß2. **(C)** Representative confocal images of the surface expression of CD11b and SIRP-ß2 on type 0 and type 1 macrophages. **(D)** Relative SIRP-ß2 mRNA expression in THP-1.EV and THP-1.SIRP-ß2 (n=2). **(E)** Representative orthogonal images of THP-1 mediated phagocytosis incubated with Daudi cells for 3h, treated with RTX (1 µg/ml). **(F)** Experimental phagocytosis of CB derived macrophages expressed EV control, SIRP-ß2 and SIRP-ß2.K202L co-cultured with SUDHL-6 and Ramos, 3h (n=3)

Suppl. Fig. 2

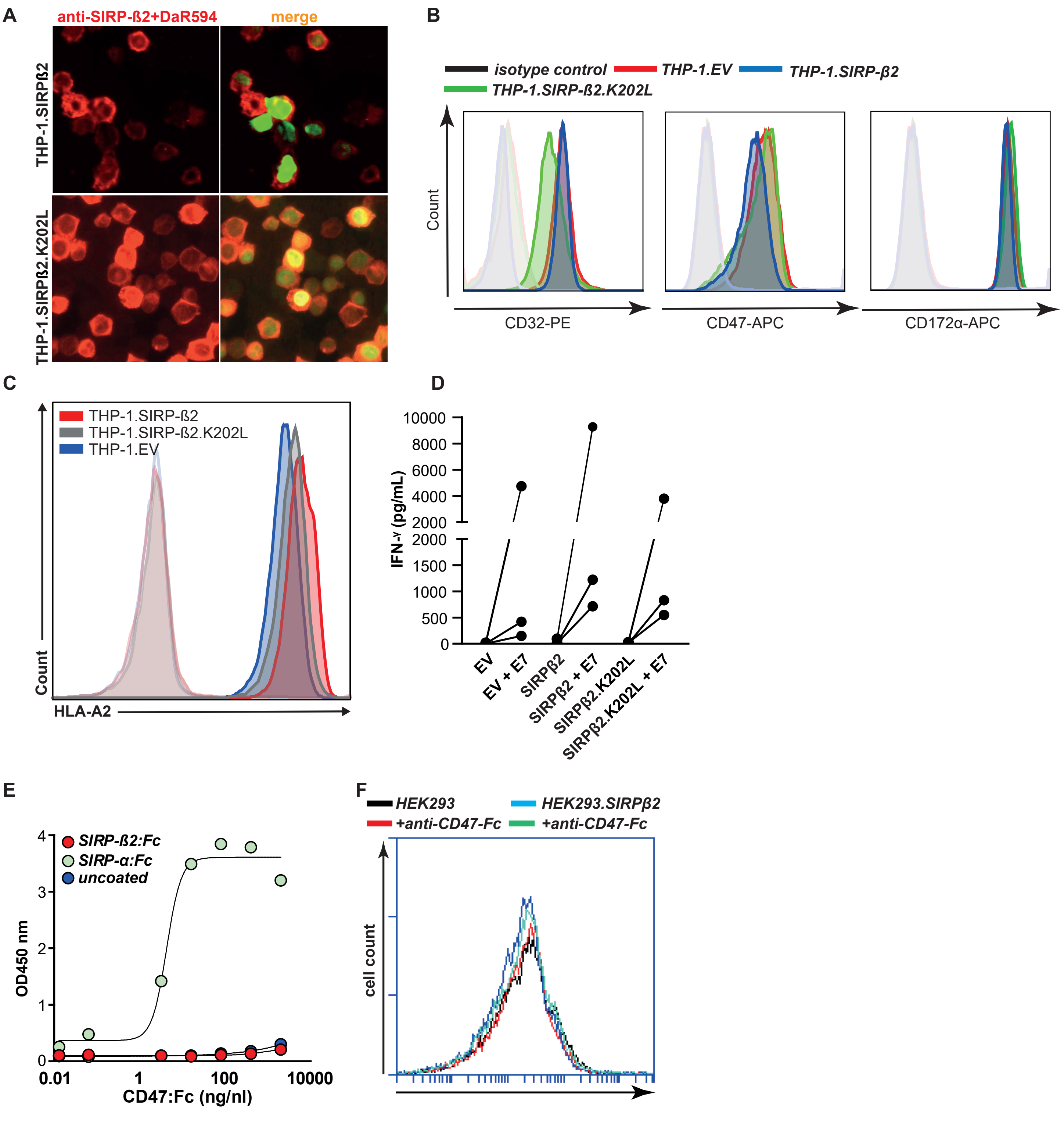

Suppl. Fig 2. Interactome analyses of SIRP-ß2 identified DAP12 as signaling component and a strong association of SIRP-ß2 with MHC-class I and potentiate HPV-E7-specific T cell responses. (A) Representative confocal microscopy images of SIRP-ß2 surface expression on THP-1.SIRP-ß2 and THP-1.SIRP-ß2.K202L. **(B)** Cell surface expression of CD32, CD47 and CD172a of THP-1.EV, THP-1.SIRP-ß2 and THP-1.SIRP-ß2.K202L, determined by flow. **(C)** HLA-ABC expression of THP-1.EV, THP-1.SIRP-ß2 and THP-1.SIRP-ß2.K202L determined by flow cytometry. **(D)** Quantitative IFN-γ secretion by primary E7-TCR specific T cells in co-culture with THP-1.EV, THP-1.SIRP-ß2 and THP-1.SIRP-ß2.K202L and with or without E7 peptide for 48 h (n=3). **(E)** Potential binding of the known SIRP-α receptor CD47 was evaluated by detecting binding of CD47:Fc on SIRP-ß2:Fc and SIRP-α:Fc-coated ELISA plates. **(F)** Histogram of binding of CD47:Fc to wild-type HEK293T cells and HEK293T cells transduced with SIRP-ß2.
